# Management of peritoneal dialysis *Mycobacterium abscessus* exit-site infection: A case report and literature review

**DOI:** 10.1177/11297298211015083

**Published:** 2021-05-13

**Authors:** Korey Bartolomeo, Mohamed Hassanein, Tushar J Vachharajani

**Affiliations:** Department of Nephrology and Hypertension, Cleveland Clinic Lerner College of Medicine of Case Western Reserve University, Glickman Urological & Kidney Institute, Cleveland Clinic, OH, USA

**Keywords:** Peritonitis, exit site infection, peritoneal dialysis, *M. abscessus*

## Abstract

Peritoneal dialysis associated infections are common and associated with high morbidity and mortality, if not treated in a timely manner. *Mycobacterium abscessus* is an uncommon pathogen in peritoneal dialysis associated infections, but is resistant to standard antimicrobial therapies used. Here we present a case of a 56 year-old male with end stage kidney disease on peritoneal dialysis for 7 years who developed a *Mycobacterium abscessus* exit-site infection. Peritonitis and peritoneal dialysis catheter tunneled line infections were ruled out and he was treated with linezolid, amikacin, and azithromycin. He required peritoneal dialysis catheter removal and hemodialysis conversion. Antibiotics were de-escalated based on *erm* inducibility and antibiotic sensitivities. Linezolid and amikacin were continued for approximately 7 total weeks, with complete resolution of the infection. Further research is needed to refine challenges in the management of *Mycobacterium abscessus* exit-site infections, including risk factors for development of *Mycobacterium abscessus*, optimal selection of empiric antibiotic therapies, duration of antibiotics, and peritoneal dialysis catheter re-insertion timing.

## Introduction

Peritoneal dialysis (PD) is a commonly employed modality for kidney replacement therapy both worldwide and in the United States. PD-associated infections include peritonitis, tunnel infections, and exit-site infections (ESI). ESI have a strong association with development of peritonitis, which frequently lead to failure of PD ultrafiltration and solute clearance, increased hospitalizations, and high mortality.^1,2^ Given this, empiric antibiotic therapies targeting the most common gram-negative and gram-positive bacteria, are used to treat suspected ESI.^3^

Nontuberculous mycobacteria are acid-fast bacilli that include *Mycobacterium abscessus* complex. *M. abscessus* are ubiquitously found in the environment, characterized by rapid growth and intrinsic resistance to common antibiotics.^4^ There is a growing body of literature of *M. abscessus* involvement in PD-associated infections with little consensus in regards to antibiotic choice, duration of treatment, and catheter removal timing.^5,6^ Publications on *M. abscessus* involved ESI in adults are more limited, with only 2 case reports from the United States.^7,8^ More recently, case reports on *M. abscessus* ESI have described differing management principles and high rates of complications, highlighting a large knowledge gap and lack of guiding evidence in this disease.^8–10^ Here we report a case of *M. abscessus* ESI and discuss current evidence supporting management of this difficult diagnosis.

## Case Report

A 56 year-old male emigrated from the Philippines 27 years ago reported purulent drainage from his PD exit site to his PD nurse in the setting of poor appetite. He has had end stage kidney disease from IgA nephropathy requiring PD for 7 years, and recurrent bacterial peritonitis from coagulase negative *Staphylococcus* (three times prior). His medical history was additionally notable for a Bosniak class IV right renal cyst, hypertension, obsessive compulsive disorder, and partially treated latent *Mycobacterium tuberculosis* 2 years prior to presentation. He was also briefly converted to hemodialysis after requiring PD catheter removal and reinsertion for *Staphylococcus* peritonitis for 2 months, 1 year prior to his current presentation. His PD regimen at the time of the noted drainage was continuous cycling PD with 3 cycles, 150 min for each cycle, and 2.8 L bags of 1.5% dextrose solution, followed by a last bag and mid-day exchange for additional solute clearance.

**Figure 1.**
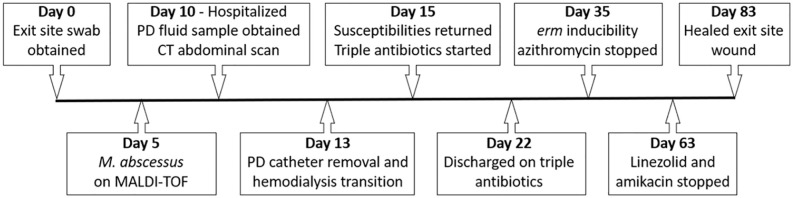
Timeline of patient’s clinical course after obtaining exit site wound culture. MALDI-TOF is matrix assisted laser desorption ionization-time of flight mass spectrometry. Triple antibiotics refers to azithromycin, amikacin, and linezolid.

His clinical course is summarized in Figure 1. Wound swabs were taken of the exit-site discharge, hypertonic saline soaks were started and he was instructed to follow up with infectious disease clinic for further management. He developed worsening nausea, vomiting, and dizziness over the next 10 days and was instructed to present to the emergency department. His initial wound swabs were identified as *Mycobacterium abscessus* on matrix assisted laser desorption ionization-time of flight (MALDI-TOF) mass spectrometry 5 days after the cultures were obtained. His initial physical examination was without abdominal tenderness or active PD exit-site drainage and his vital signs were within normal limits. His admission labs were notable for a WBC of 10.4 × 10^6^ cells/L (normal 3.7 × 10^6^–11.0 × 10^6^ cells/L) only. A PD fluid sample was obtained showing clear fluid and a total nucleated cell count of 2 cells/µL with negative gram stain and culture. Computed tomography imaging of the abdomen was negative for abscess or fluid collection.

He was continued on PD for dialysis until the catheter was removed on hospital day 3. He was transitioned to hemodialysis via a previously placed left upper extremity brachiocephalic fistula. Azithromycin 250 mg daily, linezolid 600 mg daily, and amikacin 350 mg after hemodialysis were started on hospital day 5 while genetic susceptibility tests were pending. He was discharged on hospitalization day 13 on these antibiotics, 22 days after the initial wound culture was obtained.

He was re-hospitalized 2 weeks later with community acquired pneumonia, requiring addition of piperacillin-tazobactam and transition to amoxicillin-clavulanate for 5 days. Azithromycin was also discontinued after *erm* inducibility was detected in his initial *M. abscessus* wound swab and wound swab antibiotic sensitivities were finalized (Table 1). He had one additional hospitalization for worsening dyspnea and a negative cardiac work-up for ischemia. He completed seven total weeks of linezolid and amikacin, with both discontinued after he had persistence of vestibular symptoms, including dizziness. He has since remained asymptomatic with a healed exit site wound. He has remained on hemodialysis since removal of the PD catheter.

**Table 1.** *Mycobacterium abscessus* antimicrobial susceptibilities from initial PD exit site swab.

Antibiotic	Susceptibility
Amikacin	Susceptible
Cefoxitin	Intermediate
Ciprofloxacin	Resistant
Clarithromycin	Resistant
Doxycycline	Resistant
Imipenem	Intermediate*
Linezolid	Susceptible
Moxifloxacin	Resistant
Tigecycline	No interpretation
Trimethoprim-Sulfamethoxazole	Resistant

* Imipenem results do not predict meropenem or ertapenem resistance.

## Discussion

*M. abscessus* PD ESI are likely underdiagnosed in clinical practice and underrepresented in published literature. To our knowledge this is only the third *M. abscessus* PD ESI acquired in the United States in adults, and the first without diabetes or active immunosuppression.^7,8^ Management of diagnosed *M. abscessus* ESI is challenging because of a lack of guidelines on initiation and duration of antibiotics, as well as intrinsic resistance of the pathogen.^3^ Rapid growth of *M. abscessus* allows for identification of the pathogen by day 5 after cultures taken, identified in our case based on MALDI-TOF mass spectrometry.

Once the pathogen is identified, antibiotics should be started or changed to empiric regimens for *M. abscessus*, mostly guided by in vitro susceptibilities. The most common regimens include a combination of macrolides (clarithromycin or azithromycin), aminoglycosides (amikacin), and cephalosporins (cefoxitin).^4^ Azithromycin is generally the preferred macrolide used as it was thought to have lower rates of induction of ribosomal methylase, encoded by *erm*(41), in *M. abscessus* than clarithromycin, though this has been called into question more recently.^11^ Meanwhile, with cefoxitin resistance, oxazolidinones like linezolid are considered empirically and have been used successfully in *M. abscessus* PD-associated infections, as there is moderate in vitro susceptibility, immunomodulation, clearance via multiple organs, and an oral formulation ideal for long term use.^12^

The ideal duration of multidrug antibiotics in *M. abscessus* PD-associated infections is unknown, particularly in ESI. *M. abscessus* intrinsic antibiotic resistance and recurrence of infection after cessation of antibiotics necessitates urgent PD catheter removal.^3^ Recently, many case reports have described recurrence of *M. abscessus* ESI or extension into the PD tunnel track when PD catheter salvage or PD removal and reposition techniques were used instead of complete removal.^8–10^ The PD catheter removal date should then be considered the “start” date for these antibiotic regimens. In previously diagnosed *M. abscessus* ESI without tunnel or peritonitis involvement, treatment duration on multi-drug therapies has ranged from 6 to 28 weeks.^13^ Our patient was on a total of 3 weeks of azithromycin and 7 weeks of both linezolid and amikacin, with complete resolution of his infection.

Our case highlights an unusual cause of ESI in PD as well as some of the challenges associated in management, including catheter removal, antibiotic selection, and duration. Further research is needed to refine these challenges in the management of *M. abscessus* ESI, including risk factors for development of *M. abscessus*, optimal selection of empiric antibiotic therapies, duration of antibiotics, and PD catheter re-insertion timing.
